# Supplementary material for: COVID-19 Pandemic Related Research in Africa: Bibliometric Analysis of Scholarly Output, Collaborations and Scientific Leadership
Source: Int J Environ Res Public Health. 2021 Jul 7;18(14):7273. doi: 10.3390/ijerph18147273 (PMC8308093; doi:10.3390/ijerph18147273)
Supplement: Supplementary file 1 [file ijerph-18-07273-s001.zip › ijerph-1226533-CA-supplementary.pdf]

International Journal of
Environmental Research and Public Health

MDPI

Article

Supplementary Materials

COVID-19 Pandemic Related Research in Africa: Bibliometric Analysis of Scholarly Output, Collaborations and Scientific Leadership

Maxime Descartes Mbogning Fonkou [1,†], Nicola Luigi Bragazzi [2,†], Emmanuel Kagning Tsinda [3], Yagai Bouba [4], Gideon Sadikiel Mmbando [5] and Jude Dzevela Kong [6,*]

[1] UFR IM2AG, Université Grenoble Alpes, 38000 Grenoble, France; dmbogning15@gmail.com
[2] Laboratory for Industrial and Applied Mathematics (LIAM), Department of Mathematics and Statistics, York University, Toronto, ON M3J 1P3, Canada; robertobragazzi@gmail.com
[3] Graduate School of Medicine, University of Tohoku, Sendai 980-8575, Japan; kagningemmanuel@gmail.com
[4] Chantal BIYA International Reference Centre for Research on HIV/AIDS Prevention and Management (CIRCB), Yaoundé 3077, Cameroon; romeobouba@yahoo.fr
[5] Graduate School of Life Sciences, University of Tohoku, Sendai 980-8577, Japan; gideonmmbando@gmail.com
[6] Canadian Centre for Disease Modelling (CCDM), Department of Mathematics and Statistics, York University, Toronto, ON M3J 1P3, Canada
* Correspondence: jdkong@yorku.ca; Tel.: +1-(416)-736-2100 (ext. 66093)
† Equally contributed as first authors.

1. Supplementary Data S1

1.1. Search Terms:

1.1.1. African Countries and Main Cities:

("Algeria" OR "Algerien" OR "Algerie" OR "Algiers" OR "Alger" OR "Angola" OR "Bangui" OR "Republique centrafricaine" OR "Central African Republic" OR "Benin" OR "Dahomey" OR "Benin" OR "Porto-Novo" OR "Botswana" OR "Kalahari" OR "Gaborone" OR "Burkina Faso" OR "Burkina" OR "Ouagadougou" OR "Banfora" OR "Bobo Dioulasso" OR "Burundi" OR "Bujumbura" OR "Gitega" OR "Cameroon" OR "Cameroun" OR "Yaounde" OR "Douala" OR "Bafoussam" OR "Bamenda" OR "Garoua" OR "Maroua" OR "Cape Verde" OR "Cabo Verde" OR "Praia" OR "Chad" OR "Tchad" OR "N'Djamena" OR "Tschad" OR "Comoros" OR "Comores" OR "Congo" OR "Republic of the Congo" OR "Kongo" OR "Brazzaville" OR "Cote d'Ivoire" OR "Ivory Coast" OR "Cote d'Ivoire" OR "Yamoussoukro" OR "Abidjan" OR "Bouake" OR "Korhogo" OR "Democratic Republic of the Congo" OR "Kinshasa" OR "Zaire" OR "Republique democratique du Congo" OR "Djibouti" OR "Egypt" OR "Malawi" OR "Cairo" OR "Al Minya" OR "Equatorial Guinea" OR "Guinee equatoriale" OR "Malabo" OR "Eritrea" OR "Asmara" OR "Ethiopia" OR "Ethiopie" OR "Addis Ababa" OR "Gabon" OR "Gabonese Republic" OR "Libreville" OR "Gambia" OR "Gambie" OR "Banjul" OR "Ghana" OR "Accra" OR "Takoradi" OR "Sekondi" OR "Koforidua" OR "Obuasi" OR "Kumasi" OR "Tamale" OR "Republic of Guinea" OR "Guinea" OR "Guinee" OR "Conakry" OR "Guinea-Bissau" OR "Guine-Bissau" OR "Bissau" OR "Kenya" OR "Kenia" OR "Nairobi" OR "Mombasa" OR "Lesotho" OR "Maseru" OR "Liberia" OR "Republic of Liberia" OR "Liberia" OR "Libya" OR "Libya" OR "Libye" OR "Libia" OR "Madagascar" OR "Madagaskar" OR "Madagasikara" OR "Antananarivo" OR "Toliara" OR "Fianarantsoa" OR "Antsirabe" OR "Toamasina" OR "Mahajanga" OR "Malawi" OR "Malawi" OR "Lilongwe" OR "Mali" OR "Bamako" OR "Sikasso" OR "Koutiala" OR "Koulikoro" OR "Segou" OR "Kayes" OR "Mopti" OR

"Mauritania" OR "Mauritanie" OR "Nouakchott" OR "Mauritius" OR "Ile Maurice" OR "Morocco" OR "Morocco" OR "Maroc" OR "Marruecos" OR "Rabat" OR "casablanca" OR "Mozambique" OR "Mosambik" OR "Mocambique" OR "Maputo" OR "Namibia" OR "Namibie" OR "Windhoek" OR "Niger" OR "Niamey" OR "Maradi" OR "Zinder" OR "Tillaberi" OR "Tahoua" OR "Agadez" OR "Nigeria" OR "Abuja" OR "Rwanda" OR "Ruanda" OR "Kigali" OR "Sao Tome" OR "Sao Tome" OR "Senegal" OR "Senegal" OR "Dakar" OR "Ziguinchor" OR "Seychelles" OR "Sierra Leone" OR "Somalia" OR "Somalie" OR "Mogadishu" OR "South Africa" OR "Afrique du Sud" OR "Pretoria" OR "Stellenbosch" OR "Cape Town" OR "Johannesburg" OR "South Sudan" OR "Sudsudan" OR "Juba" OR "Sudan" OR "Soudan" OR "Khartoum" OR "Swaziland" OR "eSwatini" OR "Mbabane" OR "Tanzania" OR "Zanzibar" OR "Tanganyika" OR "Tansania" OR "Tanzanie" OR "Dodoma" OR "Togo" OR "Lome" OR "Tunisia" OR "Tunesien" OR "Tunisie" OR "Tunis" OR "Gabes" OR "Uganda" OR "Ouganda" OR "Kampala" OR "Zambia" OR "Rhodesia" OR "Sambia" OR "Zambie" OR "Lusaka" OR "Zimbabwe" OR "Simbabwe" OR "Harare").

1.1.2. Terms Related to COVID:

"sars cov 2" OR "covid" OR "COVID 19" OR "COVID 19" OR "ncov" OR "2019 ncov" OR "2019nCoV" OR "novel coronavirus" OR "COVID 19" OR "covid19" OR "covid" OR "new coronavirus" OR "new corona virus" OR "novel corona virus" OR ("Wuhan" AND ("coronavirus" OR "coronavirus" OR "coronaviruses")) OR "sars cov" OR "Coronavirus Disease" OR "SARS-Cov2" OR "Coronavirus 2" OR "2019 coronavirus" OR "coronavirus 2019" OR "Corona Virus Disease" OR "SARS coronavirus" OR "Coronavirus pandemic" OR "coronavirus outbreak*" OR "china corona*" OR "coronavirus" OR "coronaviruses" OR "corona virus".

1.2. Search Queries for Each Bibliographic Database:

1.2.1. WOS Search Query

#**3**	#1 AND #2<br>Databases = WOS, CCC, KJD, MEDLINE, RSCI, SCIELO Timespan = 2019–2021<br>Search language = Auto
#**2**	AD = ("Algeria" OR "Algerien" OR "Algerie" OR "Algiers" OR "Alger" OR "Angola" OR "Bangui" OR "Republique centrafricaine" OR "Central African Republic" OR "Benin" OR "Dahomey" OR "Benin" OR "Porto-Novo" OR "Botswana" OR "Kalahari" OR "Gaborone" OR "Burkina Faso" OR "Burkina" OR "Ouagadougou" OR "Banfora" OR "Bobo Dioulasso" OR "Burundi" OR "Bujumbura" OR "Gitega" OR "Cameroon" OR "Cameroun" OR "Yaounde" OR "Douala" OR "Bafoussam" OR "Bamenda" OR "Garoua" OR "Maroua" OR "Cape Verde" OR "Cabo Verde" OR "Praia" OR "Chad" OR "Tchad" OR "N'Djamena" OR "Tschad" OR "Comoros" OR "Comores" OR "Congo" OR "Republic of the Congo" OR "Kongo" OR "Brazzaville" OR "Cote d'Ivoire" OR "Ivory Coast" OR "Cote d'Ivoire" OR "Yamoussoukro" OR "Abidjan" OR "Bouake" OR "Korhogo" OR "Democratic Republic of the Congo" OR "Kinshasa" OR "Zaire" OR "Republique democratique du Congo" OR "Djibouti" OR "Egypt" OR "Malawi" OR "Cairo" OR "Al Minya" OR "Equatorial Guinea" OR "Guinee equatoriale" OR "Malabo" OR "Eritrea" OR "Asmara" OR "Ethiopia" OR "Ethiopie" OR "Addis Ababa" OR "Gabon" OR "Gabonese Republic" OR "Libreville" OR "Gambia" OR "Gambie" OR "Banjul" OR "Ghana" OR "Accra" OR "Takoradi" OR "Sekondi" OR "Koforidua" OR "Obuasi" OR "Kumasi" OR "Tamale" OR "Republic of Guinea" OR "Guinea" OR "Guinee" OR "Conakry" OR "Guinea-Bissau" OR "Guine-Bissau" OR "Bissau" OR "Kenya" OR "Kenia" OR "Nairobi" OR "Mombasa" OR "Lesotho" OR "Maseru" OR "Liberia" OR "Republic of Liberia" OR "Liberia" OR "Libya" OR "Libya" OR "Libye" OR "Libia" OR "Madagascar" OR "Madagaskar" OR "Madagasikara" OR "Antananarivo" OR "Toliara" OR "Fianarantsoa" OR "Antsirabe" OR "Toamasina" OR "Mahajanga" OR "Malawi" OR "Malawi" OR "Lilongwe" OR "Mali" OR "Bamako" OR "Sikasso" OR "Koutiala" OR "Koulikoro" OR "Segou" OR "Kayes" OR "Mopti" OR "Mauritania" OR "Mauritanie" OR "Nouakchott" OR "Mauritius" OR "Ile Maurice" OR "Morocco" OR "Morocco" OR "Maroc" OR "Marruecos" OR "Rabat" OR "casablanca" OR "Mozambique" OR "Mosambik" OR "Mocambique" OR "Maputo" OR "Namibia" OR "Namibie" OR "Windhoek" OR "Niger" OR "Niamey" OR "Maradi" OR "Zinder" OR "Tillaberi" OR "Tahoua" OR "Agadez" OR "Nigeria" OR "Abuja" OR "Rwanda" OR "Ruanda" OR "Kigali" OR "Sao Tome" OR "Sao Tome" OR "Senegal" OR "Senegal" OR "Dakar" OR "Ziguinchor" OR "Seychelles" OR "Sierra Leone" OR "Somalia" OR "Somalie" OR "Mogadishu" OR "South Africa" OR "Afrique du Sud" OR "Pretoria" OR "Stellenbosch" OR "Cape Town" OR "Johannesburg" OR "South Sudan" OR "Sudsudan" OR "Juba" OR "Sudan" OR "Soudan" OR "Khartoum" OR "Swaziland" OR "eSwatini" OR "Mbabane" OR "Tanzania" OR "Zanzibar" OR "Tanganyika" OR "Tansania" OR "Tanzanie" OR "Dodoma" OR "Togo" OR "Lome" OR "Tunisia" OR "Tunesien" OR "Tunisie" OR "Tunis" OR "Gabes" OR "Uganda" OR "Ouganda" OR "Kampala" OR "Zambia" OR "Rhodesia" OR "Sambia" OR "Zambie" OR "Lusaka" OR "Zimbabwe" OR "Simbabwe" OR "Harare")<br>Databases = WOS, CCC, KJD, MEDLINE, RSCI, SCIELO Timespan = 2019–2021<br>Search language = Auto
#**1**	TI = ("sars cov 2" OR "covid" OR "COVID 19" OR "COVID 19" OR "ncov" OR "2019 ncov" OR "2019nCoV" OR "novel coronavirus" OR "COVID 19" OR "covid19" OR "covid" OR "new coronavirus" OR "new corona virus" OR "novel corona virus" OR ("Wuhan" AND ("coronavirus" OR "coronavirus" OR "coronaviruses")) OR "sars cov" OR "Coronavirus Disease" OR "SARS-Cov2" OR "Coronavirus 2" OR "2019 coronavirus" OR "coronavirus 2019" OR "Corona Virus Disease" OR "SARS coronavirus" OR "Coronavirus pandemic" OR "coronavirus outbreak*" OR "china corona*" OR "coronavirus" OR "coronaviruses" OR "corona virus")<br>Databases = WOS, CCC, KJD, MEDLINE, RSCI, SCIELO Timespan = 2019–2021<br>Search language = Auto

1.2.2. PubMed Search Query

#**3**	#1 AND #2
#**2**	Search: "sars cov 2"[Title] OR "covid"[Title] OR "COVID 19"[Title] OR "COVID 19"[Title] OR "ncov"[Title] OR "2019 ncov"[Title] OR "2019nCoV"[Title] OR "novel coronavirus"[Title] OR "COVID 19"[Title] OR "covid19"[Title] OR "covid"[Title] OR "new coronavirus"[Title] OR "new corona virus"[Title] OR "novel corona virus"[Title] OR ("Wuhan"[Title] AND ("coronavirus"[Title] OR "coronavirus"[Title] OR "coronaviruses"))[Title] OR "sars cov"[Title] OR "Coronavirus Disease"[Title] OR "SARS-Cov2"[Title] OR "Coronavirus 2"[Title] OR "2019 coronavirus"[Title] OR "coronavirus 2019"[Title] OR "Corona Virus Disease"[Title] OR "SARS coronavirus"[Title] OR "Coronavirus pandemic"[Title] OR "coronavirus outbreak*"[Title] OR "china corona*"[Title] OR "coronavirus"[Title] OR "coronaviruses"[Title] OR "corona virus"[Title] Filters: from 2019/12 - 2021/3 Sort by: Publication Date
#**1**	Search: "Algeria"[Affiliation] OR "Algerien"[Affiliation] OR "Algerie"[Affiliation] OR "Algiers"[Affiliation] OR "Alger"[Affiliation] OR "Angola"[Affiliation] OR "Bangui"[Affiliation] OR "Republique centrafricaine"[Affiliation] OR "Central African Republic"[Affiliation] OR "Benin"[Affiliation] OR "Dahomey"[Affiliation] OR "Benin"[Affiliation] OR "Porto-Novo"[Affiliation] OR "Botswana"[Affiliation] OR "Kalahari"[Affiliation] OR "Gaborone"[Affiliation] OR "Burkina Faso"[Affiliation] OR "Burkina"[Affiliation] OR "Ouagadougou"[Affiliation] OR "Banfora"[Affiliation] OR "Bobo Dioulasso"[Affiliation] OR "Burundi"[Affiliation] OR "Bujumbura"[Affiliation] OR "Gitega"[Affiliation] OR "Cameroon"[Affiliation] OR "Cameroun"[Affiliation] OR "Yaounde"[Affiliation] OR "Douala"[Affiliation] OR "Bafoussam"[Affiliation] OR "Bamenda"[Affiliation] OR "Garoua"[Affiliation] OR "Maroua"[Affiliation] OR "Cape Verde"[Affiliation] OR "Cabo Verde"[Affiliation] OR "Praia"[Affiliation] OR "Chad"[Affiliation] OR "Tchad"[Affiliation] OR "N'Djamena"[Affiliation] OR "Tschad"[Affiliation] OR "Comoros"[Affiliation] OR "Comores"[Affiliation] OR "Congo"[Affiliation] OR "Republic of the Congo"[Affiliation] OR "Kongo"[Affiliation] OR "Brazzaville"[Affiliation] OR "Cote d'Ivoire"[Affiliation] OR "Ivory Coast"[Affiliation] OR "Cote d'Ivoire"[Affiliation] OR "Yamoussoukro"[Affiliation] OR "Abidjan"[Affiliation] OR "Bouake"[Affiliation] OR "Korhogo"[Affiliation] OR "Democratic Republic of the Congo"[Affiliation] OR "Kinshasa"[Affiliation] OR "Zaire"[Affiliation] OR "Republique democratique du Congo"[Affiliation] OR "Djibouti"[Affiliation] OR "Egypt"[Affiliation] OR "Malawi"[Affiliation] OR "Cairo"[Affiliation] OR "Al Minya"[Affiliation] OR "Equatorial Guinea"[Affiliation] OR "Guinee equatoriale"[Affiliation] OR "Malabo"[Affiliation] OR "Eritrea"[Affiliation] OR "Asmara"[Affiliation] OR "Ethiopia"[Affiliation] OR "Ethiopie"[Affiliation] OR "Addis Ababa"[Affiliation] OR "Gabon"[Affiliation] OR "Gabonese Republic"[Affiliation] OR "Libreville"[Affiliation] OR "Gambia"[Affiliation] OR "Gambie"[Affiliation] OR "Banjul"[Affiliation] OR "Ghana"[Affiliation] OR "Accra"[Affiliation] OR "Takoradi"[Affiliation] OR "Sekondi"[Affiliation] OR "Koforidua"[Affiliation] OR "Obuasi"[Affiliation] OR "Kumasi"[Affiliation] OR "Tamale"[Affiliation] OR "Republic of Guinea"[Affiliation] OR "Guinea"[Affiliation] OR "Guinee"[Affiliation] OR "Conakry"[Affiliation] OR "Guinea-Bissau"[Affiliation] OR "Guine-Bissau"[Affiliation] OR "Bissau"[Affiliation] OR "Kenya"[Affiliation] OR "Kenia"[Affiliation] OR "Nairobi"[Affiliation] OR "Mombasa"[Affiliation] OR "Lesotho"[Affiliation] OR "Maseru"[Affiliation] OR "Liberia"[Affiliation] OR "Republic of Liberia"[Affiliation] OR "Liberia"[Affiliation] OR "Libya"[Affiliation] OR "Libya"[Affiliation] OR "Libye"[Affiliation] OR "Libia"[Affiliation] OR "Madagascar"[Affiliation] OR "Madagaskar"[Affiliation] OR "Madagasikara"[Affiliation] OR "Antananarivo"[Affiliation] OR "Toliara"[Affiliation] OR "Fianarantsoa"[Affiliation] OR "Antsirabe"[Affiliation] OR "Toamasina"[Affiliation] OR "Mahajanga"[Affiliation] OR "Malawi"[Affiliation] OR "Malawi"[Affiliation] OR "Lilongwe"[Affiliation] OR "Mali"[Affiliation] OR "Bamako"[Affiliation] OR "Sikasso"[Affiliation] OR "Koutiala"[Affiliation] OR "Koulikoro"[Affiliation] OR "Segou"[Affiliation] OR "Kayes"[Affiliation] OR

	"Mopti"[Affiliation] OR "Mauritania"[Affiliation] OR "Mauritanie"[Affiliation] OR "Nouakchott"[Affiliation] OR "Mauritius"[Affiliation] OR "Ile Maurice"[Affiliation] OR "Morocco"[Affiliation] OR "Morocco"[Affiliation] OR "Maroc"[Affiliation] OR "Marruecos"[Affiliation] OR "Rabat"[Affiliation] OR "casablanca"[Affiliation] OR "Mozambique"[Affiliation] OR "Mosambik"[Affiliation] OR "Mocambique"[Affiliation] OR "Maputo"[Affiliation] OR "Namibia"[Affiliation] OR "Namibie"[Affiliation] OR "Windhoek"[Affiliation] OR "Niger"[Affiliation] OR "Niamey"[Affiliation] OR "Maradi"[Affiliation] OR "Zinder"[Affiliation] OR "Tillaberi"[Affiliation] OR "Tahoua"[Affiliation] OR "Agadez"[Affiliation] OR "Nigeria"[Affiliation] OR "Abuja"[Affiliation] OR "Rwanda"[Affiliation] OR "Ruanda"[Affiliation] OR "Kigali"[Affiliation] OR "Sao Tome"[Affiliation] OR "Sao Tome"[Affiliation] OR "Senegal"[Affiliation] OR "Senegal"[Affiliation] OR "Dakar"[Affiliation] OR "Ziguinchor"[Affiliation] OR "Seychelles"[Affiliation] OR "Sierra Leone"[Affiliation] OR "Somalia"[Affiliation] OR "Somalie"[Affiliation] OR "Mogadishu"[Affiliation] OR "South Africa"[Affiliation] OR "Afrique du Sud"[Affiliation] OR "Pretoria"[Affiliation] OR "Stellenbosch"[Affiliation] OR "Cape Town"[Affiliation] OR "Johannesburg"[Affiliation] OR "South Sudan"[Affiliation] OR "Sudsudan"[Affiliation] OR "Juba"[Affiliation] OR "Sudan"[Affiliation] OR "Soudan"[Affiliation] OR "Khartoum"[Affiliation] OR "Swaziland"[Affiliation] OR "eSwatini"[Affiliation] OR "Mbabane"[Affiliation] OR "Tanzania"[Affiliation] OR "Zanzibar"[Affiliation] OR "Tanganyika"[Affiliation] OR "Tansania"[Affiliation] OR "Tanzanie"[Affiliation] OR "Dodoma"[Affiliation] OR "Togo"[Affiliation] OR "Lome"[Affiliation] OR "Tunisia"[Affiliation] OR "Tunesien"[Affiliation] OR "Tunisie"[Affiliation] OR "Tunis"[Affiliation] OR "Gabes"[Affiliation] OR "Uganda"[Affiliation] OR "Ouganda"[Affiliation] OR "Kampala"[Affiliation] OR "Zambia"[Affiliation] OR "Rhodesia"[Affiliation] OR "Sambia"[Affiliation] OR "Zambie"[Affiliation] OR "Lusaka"[Affiliation] OR "Zimbabwe"[Affiliation] OR "Simbabwe"[Affiliation] OR "Harare"[Affiliation] Filters: from 2019/12 - 2021/3 Sort by: Publication Date

1.2.3. AJOL Search Query

(covid OR coronavirus) AND Africa

Table S1. COVID-19 research collaboration between African countries and non-African countries.

		Central Africa							Eastern Africa														North Africa						Southern Africa										Western Africa														
		Cameroon	Central African Rep	Chad	Congo	D R Congo	Equatorial Guinea	Gabon	Burundi	Comoros	Eritrea	Ethiopia	Kenya	Madagascar	Mauritius	Rwanda	Seychelles	Somalia	South Sudan	Sudan	Tanzania	Uganda	Algeria	Djibouti	Egypt	Libya	Morocco	Tunisia	Angola	Botswana	Lesotho	Malawi	Mozambique	Namibia	South Africa	Swaziland	Zambia	Zimbabwe	Benin	Burkina Faso	Ghana	Guinea	Guinea-Bissau	Ivory Coast	Liberia	Mali	Mauritania	Niger	Nigeria	Senegal	Sierra Leone	The Gambia	Togo
East Asia & Pacific	Australia	13			1	3	1	2				13	27	1	3	2				3	10	14	4		32	4	11	5		4	1	6	5	2	158	1	7	7	2	2	27	1	3	2			1	1	47	3	3	2	
	Brunei																																																1				
	Cambodia	10				2						1											1		1	1	1						1		2				1	2	1	1	1			1			2	1			
	China	7	1		6	2	1	4			1	7	22	2	3	2			1	12	10	13	3		94	1	14	12		1		6	5	2	113		10	2	2	3	26			3		1	1	1	56	8	1	1	1
	Indonesia				1	1	1						5					1		1	2	4	1		20	4	4	8				1	1		10		1	3			2								17				
	Japan	1			1	1						6	8			2				6	2	6	4		55	4	11	1			1	1	3	1	47		5	1		2	8	1		2					20	1		1	
	Laos	2											1								1												1																	1			
	Malaysia	2						1				1	7		1			1		2	2	7	4		27	2	6	5		1		3	2	2	26	1	2	3			12	1							22	2			
	Mongolia											1													2																												
	Myanmar												6								1	2			5	4							1		3			1											5				
	New Zealand											1	4							6		3			17		2	11			1	1	4		31		1		1	1				1					16	2		1	
	Papua New Guinea												4								2	3									2	2	1		3														3			2	
	Philippines	1			2	1							4			3				1	1	5	3		10		4	1				1	2	1	10		1	1	1		2	1		1				1	7	1	1		
	Singapore	2			1	1		2				3	6								2	5	1		19		2	6					4		37		2	5			5			2				1	17	2	1	2	
	South Korea											4	6	1		1				1	1	2	2		27	5	6	5					1		31		4	1			4			1					14	3			
	Thailand	1			1	1		1				5	9	2		1		1			3	4	2		16	5	3	1					3		14		1	1			2								26	6			
	Vietnam	1			1	1		4				1	5		1			1		3	1	4	2		11		4			1		1	4	1	14	1	1	3			4								9	5	1		
Europe & Central Asia	Albania												1								1	1			3		1	1					1		1			1											1				
	Armenia																			2		1	1		3		1	1									1	1											1				
	Austria							1					6							5	1	2			16	3		3				1	1		34			1			4			1		1			3	1		2	
	Azerbaijan																								4										2											3							
	Belarus	1																							2	1									1																		
	Belgium	10		1	1	30	1	1				6	14	1		7		3			6	14	1		11	2	6	1		2			10		80		5	7	3	4	9	5	1	4		2			16	10		2	
	Bosnia and Herzegovina												4										1		5	4	1	1													1								4				
	Bulgaria	1					1													1		1	1		7	2		1					1		3			2			2								2				
	Croatia												1										2		3	1	3	1					2		4						1								4				
	Cyprus												1												3										4														6				
	Czech Republic						1						3									1			13								1		11		1	1			1						1						
	Denmark				7			1				1	6	1	1	3					4	3			13		2	1		2		1	1		37		2	1		1	3		3	2					7	1	1		
	Estonia																								1																1								1				
	Finland	1					1						2		1	1		1		3		3			16		1	1					3		19			2											8				
	France	33	5	3	3	11		4				8	17	13	1	4	1			7	5	9	16		56	8	33	13		2		2	9		76	1	4	6	2	11	11	4		9		3	2	3	21	22	3	1	6
	Georgia											1								3	1	1	1		2		1						3		6						1								1				
	Germany	6			13	3		13				9	22	2	1	3		1		6	7	8	2		83	5	6	13		2		4	5	1	116		6	6	4	1	29	1	2	1		3			35	11	3		
	Greece	1					1						3	1		1				2		1	1		15	3	4	3					3		15						2		1						12				
	Hungary												4							1		1			5			2					1		7			1											3				
	Iceland				1																																		1				1				1			1			
	Ireland	1											6								2	6	1		13	1	3			1		1	1		27		1	2			1	1							2				
	Italy	9			6		1		1			6	26		1	5				12	6	14	7	1	102	8	12	30		2		2	9	1	119	2	5	5	1	2	15			1		3		1	38	4	6		
	Kazakhstan																								1																												
	Kosovo						1																		10			10																					12				
	Kyrgyzstan																			2													2		1																		
	Latvia																								1										1																		
	Lithuania	1										1	2							2		2			4								3		8			1														1	
	Luxembourg																				1	1			2										3														2				
	Moldova																								1										1						1												
	Monaco																																		1																		
	Montenegro																						1				1	1																									
	Netherlands	2			2	1		5				9	19	1		1		1		4	4	9	2		27	2	8	8		2			7		77		1	4		1	8	1	1	3	1			1	24	5	4	3	
	Norway	4						2				6	10	1		2		2		1	6	8	1		9	4	3	4		1		3	2		32		6	7			12			1					11	1	3	1	
	Poland	1											2							1	1	3	3		24	2		4		1		1	1	1	19	1	2	2			4								4			2	
	Portugal				2								4							2	1	1	2		25	4	3	14		2		1	7		30		1						1						19		1	1	
	North Macedonia						1																1		3		1	1							1						1												
	Romania						1															1	4		13	3		2					1		7														5				
	Russia				1		1						5			2						2	1		23	1	5	6					1		30		3	2		2	3							1	4	1	2		
	Serbia	1					1						7							1		4	3		9	5	1	1		1		2	1	2	7	1	2	1			3								10				
	Slovakia												2												4										1																1		
	Slovenia						1														1	2	1		7		2	5					2		10														1				
	Spain	2					1	1				4	13			2				5	6	4	7		57	2	15	16				2	28	1	72		3	2		1	8			2		2		1	20		2		
	Sweden	3				1						3	13			6		1		6	4	10	3		26	2	4	1		2	1	8	6	2	61	1	5	1			7			2					19	4	3	2	
	Switzerland	12	2		9	6		2				8	22	1		4		1		6	8	13	3		55	2	8	5		1		3	5	2	86		5	6	4		13		1	6	1	6	3	1	15	6	4	3	
	Turkey	1					1	1				2	5			1	1	1		7	1	5	6	1	46	2	11	11					3		30			1			5	1				4	1	1	25				1
	Ukraine																					1	1		1										5														3				
	United Kingdom	27	1		14	23		11	1		1	32	98	4	5	18		3		32	32	55	6		172	18	22	29		9	1	31	29	4	360	2	22	29	10	3	68	4	1	9	2	5	2	1	175	22	9	18	1
	Uzbekistan																								1	1																											
Latin America & the Caribbean	Antigua and Barbuda																								1																												
	Argentina	1					1					3	7			1					1	3	2		20	3	4	5		1			1		30			1			2							1	10	2	1	3	
	Aruba																									1																											
	Bahamas																								1																												
	Barbados																																								1												
	Bolivia											1	1			1							1		1	1									1														1				
	Brazil	6				2	1	1				5	18	1	1	2		1		7	3	13	3		56	1	12	22				2	12		108		4	4		1	17		1	4				1	33	3	2	4	
	Chile	1		1			1	1					6			1					2	2			9		3	3				2			29		3	1		1	4								4	1			
	Colombia	6				1	1					1	12		1				1		2	4	1		19	1	1	10							28		1			1	9			2		2			18		1		
	Costa Rica	1											1								1	2	1		3	1				1			2		3						2								2				
	Cuba												2												1										2			1												1			
	Dominica																								1																								3				
	Dominican Republic												1		1	1						1			1													1															
	Ecuador					1												2				2			2			1					1		4		1	3															
	Grenada																								1																								6				
	Guatemala	1				2						1	3	1								1						1			1	2			7		1	1							1	1			3			2	1
	Haiti					2						1	1			2																																					2
	Honduras	1																			1						1														1												
	Jamaica	1										1	4																						4														1				
	Mexico	1				1	1					1	10	3		2				4	1	3	1		23	2	6	2				1	4		32		1	2		1	1					1		1	10		1	2	
	Panama																											1							1														1				
	Peru	1			1	4	1					2	13			1		1			1	5	1		8	5	2	1			1	1	1	1	13		3	2			1					6			11	1		1	
	Saint Kitts and Nevis																								1										1																		
	Saint Lucia											1																							1																		
	Saint Vincent and the Grenadines																																																1				
	Sint Maarten																								1																												
	Suriname																										1																										
	Trinidad and Tobago																																		1		1												1				
	Uruguay	1										1	1								1				2		4	1		1					4		1			1	1								1				
	Venezuela																			1			1		3	1		1							4			1											3				
Middle East	Bahrain																			3			1		13		3	2							1			1											1				
	Iran											3	11		1	1				8	1	3	4	1	35	6	4	7					2		33			1			6	1				6	1	1	16				1
	Iraq	1											4			2				2		3	7		22		8	6		1		2		2	5	1	2	1			3								4				
	Israel	1					1						9			2				2	1	4	5		14	5	6	3					2		28		2	2		2	1								8	1			
	Jordan												4							9		1	12		51	6	11	15							3						1								6				
	Kuwait											1	1							1			8		29		8	6							1															1			
	Lebanon												3			1				5	1	3	5		31	1	7	17					1		11			2			1	1							16	1			
	Malta				1																														2				1										3	1			
	Oman				3								2					1		5			2		16	1	3	3							12		1				1			2					4				
	Qatar											2			4					3			2	1	25	3	8	8							11						1								8				
	Saudi Arabia	5			3	1		1				2	8		1					32	4	5	19	1	317	10	24	33		1		3		1	45	1	8	1	1		8					14			37	4			
	Syria												1							4		1	3		5	1	2	2					3		1														1				
	United Arab Emirates	3										2	4							5	1	1	7		50	7	12	12							11		1	2		1	3								13				
	Yemen												4							4			1	1	15	4	5	3					1		2														4				
North America	Canada	8		1	2	12						16	34			10		1		4	5	20	9		53	1	18	18		3		5	12		128		8	8	4	6	22	3	1	3	1	1			52	11	1	1	2
	United States	36	1	1	11	26	2	2	1	2		48	114	14	5	30	1	4	2	15	29	70	15		257	3	32	39		15	1	14	20	4	414	3	30	23	8	7	60	4	3	12	6	25	2	4	159	28	6	6	2
South Asia	Afghanistan					1										1				4		1			2	1	1	1						1	2			1											8				
	Bangladesh	4			1							2	10	1	1	3				3	1	7	2		22	1	1	2			1	2	3	1	16		1	5			4								17			2	
	Bhutan												2			1						1			1													1															
	India	7			4	10	1	1			3	24	25		2	2	1		1	10	9	20	5		101	6	22	24		2	1	4	8	2	114		7	2	4	3	17	2	1	1		6	1	1	68		2	3	
	Maldives											4																																									
	Nepal											3	3			1				1	1	4	2		8	1	4								7														7				
	Pakistan	2										4	9			4		1		6	4	7	6		44	3	6	6				2		1	21		4	3		1	7								32		1		
	Sri Lanka	3											4			1					1	1			4			1							3		1	1			1												

Table S2. COVID-19 research collaboration between African countries.

		Central Africa							Eastern Africa														North Africa						Southern Africa										Western Africa														
		Cameroon	Central African Republic	Chad	Congo	D R Congo	Equatorial Guinea	Gabon	Burundi	Comoros	Eritrea	Ethiopia	Kenya	Madagascar	Mauritius	Rwanda	Seychelles	Somalia	South Sudan	Sudan	Tanzania	Uganda	Algeria	Djibouti	Egypt	Libya	Morocco	Tunisia	Angola	Botswana	Lesotho	Malawi	Mozambique	Namibia	South Africa	Swaziland	Zambia	Zimbabwe	Benin	Burkina Faso	Ghana	Guinea	Guinea-Bissau	Ivory Coast	Liberia	Mali	Mauritania	Niger	Nigeria	Senegal	Sierra Leone	The Gambia	Togo
Central Africa	Cameroon	127																																																			
	Central African Republic	5	5																																																		
	Chad			5																																																	
	Congo	2	2		43																																																
	D R Congo	7	1		2	81																																															
	Equatorial Guinea						3																																														
	Gabon	1			1			22																																													
Eastern Africa	Burundi				1				5																																												
	Comoros									2																																											
	Eritrea										4																																										
	Ethiopia	5		1	2	2						322																																									
	Kenya	13			2	9				2		14	289																																								
	Madagascar	3	2		2	1		1		2			4	29																																							
	Mauritius	1											3	1	32																																						
	Rwanda	4				6						4	10			51																																					
	Seychelles													1	1		2																																				
	Somalia					1							1					12																																			
	South Sudan											1	1						4																																		
	Sudan	2			1							3	5				1			113																																	
	Tanzania	6			2	4		2				5	14			7		1		3	89																																
	Uganda	9			1	6						10	29			7		3		4	17	169																															
North Africa	Algeria	1											3		1					4			113																														
	Djibouti																							3																													
	Egypt	9			2							5	16		1	3		1		19	6	10	18	1	1220																												
	Libya												4							1			2		9	56																											
	Morocco	2										2	5		1	1		1		6	1	3	18		21		366																										
	Tunisia	1											4		1					6		4	11		28	2	12	159																									
Southern Africa	Angola																												3																								
	Botswana	2										1	4			1				1		5			1					29																							
	Lesotho												1																		3																						
	Malawi	2			1	1		1		2		3	11	3		1				1	2	7			1					3	1	57																					
	Mozambique	2				4						4	4	1	1	3	1	1		3	5	8			2		4	1		1		2	65																				
	Namibia	1											3			1				1		2			1					2		2		17																			
	South Africa	19		1	9	19		1	1			13	62	4	6	14	1			11	17	31	4		35	2	8	10		8	2	15	11	4	1452																		
	Swaziland	1										1	1							1		1			1					1		1		1	2	11																	
	Zambia	3			4	4				2		4	10	2		1				4	6	9			3		1	2		3		6		2	13	1	57																
	Zimbabwe	4			3	1		1				4	11			2				3	4	10			7			2		3		4	3	1	32	1	4	91															
Western Africa	Benin	3	1		4	2						1	2		1							1	1		1		2	1							3				27														
	Burkina Faso	3	3		4	1		2				2	5	3							1		1				2	1							3		2	1	2	35													
	Ghana	13			2	5		3	2			7	19	2	1	6				7	10	15	3		11		4	6		2		5	2	5	39	1	6	6	4	1	234												
	Guinea	4	1		1	3						1	1			1					2	3		1			1						1		1		1	1	6	5	2	21											
	Guinea-Bissau				1	1																													1				2	1		1	6										
	Ivory Coast	6	1		1	1							2			1							1				2						1		4				1	2	4	2		21									
	Liberia	1				2						1	2	1		3					1	2								1					2		1	1			2	1			9								
	Mali	2				4						1	1	1				1				1		1			2								3		5			2		3		1	1	41							
	Mauritania				2	1																		1											1				1			1	1			1	6						
	Niger	4	2		2								4	2										1	2										5			2		2		2				1	1	15					
	Nigeria	11			2	6			2			19	43	1	2	9			1	11	14	21	4		36	5	7	16		2	1	8	2	3	97	1	6	8	4	1	32	2		4	2	2		2	757				
	Senegal	8	2		1	2		4				4	6	4		2					1	1	2		2		1					1	2		14			2	3	7	7	4	1	6	1	2	1		9	88			
	Sierra Leone				1			1					3							2	3	2													5		3	2			4					1		2	5		31		
	The Gambia	1						2				1	2								1	2									1	1			5			1		1	1					1			6	1		23	
	Togo	1	1			4							1	1		2								1			1								1				1	1		2		2	1	2	1	1		1			10
